# Psychological well-being of medical and dental students in Saudi Arabia post worldwide pandemic: a cross sectional study

**DOI:** 10.1186/s12909-025-07817-0

**Published:** 2025-09-01

**Authors:** Ahmad Hassan Jabali

**Affiliations:** Department of Restorative Dental Sciences, Consultant & Associate Professor of Endodontics, College of Dentistry, Jazan University, Jazan, Saudi Arabia

**Keywords:** Depression, Anxiety, Stress, Medical students, Dental students, DASS-21, Saudi arabia

## Abstract

**Background:**

Medical and dental students face significant psychological challenges due to rigorous academic demands and clinical responsibilities, which often lead to depression, anxiety, and stress (DAS). The COVID-19 pandemic has worsened these issues internationally, and Saudi Arabia (SA) is not an exclusion. This study aimed to measure the prevalence of DAS among Saudi Arabian medical and dental students and determine significant socio-demographic and academic factors affecting their psychological well-being especially post worldwide pandemics.

**Methods:**

A cross-sectional survey was carried out among Saudi Arabian medical and dental students in July and August 2021. An online survey that included demographic information and the depression, anxiety, and stress (DASS-21) scale was used to gather data. It was disseminated nationwide via university emails and academic platforms. A total of 715 students participated in the survey, of whom 550 were dental students and 165 were medical students. Statistical analysis, including ordinal regression, was carried out using SPSS to assess the relationships between mental health outcomes and characteristics including study field, academic phase, and geography. Data were documented and statistically analyzed, and statistical significance was set at *p* < 0.05 for all parameters and tests. Cronbach’s alpha scores for all DASS-21 subscales were more than 0.87, confirming the instrument’s reliability.

**Results:**

The prevalence rates of stress, anxiety, and depression were 88.5%, 67.3%, and 51.3%, respectively. Medical students reported higher levels of extreme stress (38.2%) and anxiety (42.4%) than dental students (30.4% and 24.7%, respectively). Medical students reported considerably higher stress in their clinical phase compared with their counterparts in preclinical phase. Preclinical dental students showed higher anxiety and depression than clinical students. Socio-demographic characteristics such as living conditions and region showed significant differences (*p* < 0.05), while gender and marital status exhibited no significant differences.

**Conclusion:**

This study reveals a high prevalence of psychological distress among medical and dental students in Saudi Arabia, particularly clinical medical and preclinical dental students. These findings emphasize the need for targeted interventions to address academic stress and enhance mental well-being, emphasizing the importance of collaborative efforts to support students’ mental health and resilience.

**Supplementary Information:**

The online version contains supplementary material available at 10.1186/s12909-025-07817-0.

## Background

Medical and dental students consistently report elevated levels of psychological distress compared to peers in other academic disciplines, primarily due to the demanding and high-stakes nature of their academic and clinical training [1–4]. Prolonged exposure to stress, especially without access to effective coping mechanisms, may impair cognitive performance and undermine academic success [5, 6]. Common stressors include clinical responsibilities, time pressure, emotionally demanding patient interactions, and the persistent expectation for self-directed learning and professional excellence [7].

These pre-existing stressors were markedly intensified by the COVID-19 pandemic, which disrupted traditional academic structures and introduced unprecedented psychological burdens. Health profession students experienced abrupt transitions to remote learning, uncertainty surrounding examinations and clinical placements, and heightened fears of infection [8, 9]. Medical and dental students were particularly vulnerable, due to their limited experience managing public health emergencies and elevated clinical exposure [10–12]. Dental students faced additional risk given the aerosol-generating nature of many procedures, classifying them as high-risk by the Occupational Safety and Health Administration [13]. Region-specific studies confirmed elevated psychological burden: in India, over 75% of students reported moderate to high stress during the pandemic, especially among females aged 21–25 [12]; in Pakistan, final-year students expressed concern over their futures despite professional commitment [10]. In Saudi Arabia, elevated DAS levels were reported among dental students [11], with a longitudinal study finding that females, unmarried students, and those in early academic years were more affected [14].

The Depression, Anxiety, and Stress Scale-21 (DASS-21) was selected as the most appropriate tool for this study, due to its conciseness, robust cross-cultural validation, and ability to measure all three psychological domains concurrently [15]. In contrast to single-domain instruments such as the Beck Anxiety Inventory [16], GAD-7 [17, 18], or the Perceived Stress Scale [19], which either lack severity grading or ignore symptom overlap, DASS-21 offers nuanced differentiation between co-occurring emotional states. Curriculum-specific tools like the Dental Environment Stress Questionnaire [20] also fall short in capturing the broader psychological landscape. DASS-21 aligns with the tripartite model of emotional disorders [21], enabling differentiation between shared and distinct features of DAS, making it well-suited for research in academic health settings [22, 23].

Sociodemographic and behavioral factors such as gender, academic year, income, sleep, and physical activity have been widely studied in relation to depression, anxiety, and stress (DAS) among health profession students. While several studies suggest that female and preclinical students report higher psychological distress [1–3, 6, 24], others have found no significant associations with gender or academic year [25–29], reflecting inconsistent trends. For instance, Abdulghani et al. (2011) and Naz et al. (2017) found that distress levels were notably higher among female and early-year students [6, 30], while Sravani et al. (2018) reported a peak in third-year dental students coinciding with the onset of clinical exposure [31]. In broader contexts, de Oliveira Viana et al. (2024) identified low income and physical inactivity as predictors of DAS among Brazilian dental students [32], and Malik et al. (2024) linked psychological distress to impaired academic performance in medical students [33]. Yusoff et al. (2010) emphasized academic workload as a key stressor [34], while local studies by Basudan et al. (2017) and Nayak & Sahu (2021) highlighted the roles of gender, sleep duration, and communication issues [35, 36]. A systematic review by Moradi et al. (2024) further confirmed high DAS and sleep disturbance rates among dental students, underscoring the urgency of multifaceted mental health interventions in academic settings [37].

Although several Saudi-based studies have explored DAS among health students, most were limited to single institutions, specific disciplines, or early pandemic periods. Thus, national-level data that examines medical and dental students across academic years in the post-pandemic context remains scarce. Moreover, inconsistent findings regarding the impact of gender and academic stage highlight the need for robust, comparative studies using standardized tools.

This study aims to address these gaps by evaluating the psychological well-being of medical and dental students across Saudi Arabia in the aftermath of the COVID-19 pandemic. Using the validated DASS-21 scale, it investigates how demographic, academic, and lifestyle factors influence DAS levels. Based on previous research. It was hypothesized that differences in psychological distress levels would exist between students based on their academic phase and field of study.

## Methods

### Study design, setting, and ethical approval

A cross-sectional study was conducted to evaluate the mental wellness of medical and dental students in the Kingdom of Saudi Arabia during July and August 2021. Data were collected using a nationwide online Google Forms questionnaire, distributed through official university emails and a reminder through widely used other platforms for reminding the participants including WhatsApp. Approval was granted by the Ethical Committee, Jazan University (Reference Number: CODJU-2118 F). Participants provided informed consent electronically, with assurances of anonymity and option to withdraw at any time. Data were handled according to data protection regulations.

### Sample size calculation

A complete list of dental and medical students was obtained from the Saudi Commission for Health Specialties. The sample size was determined to ensure representativeness across each region. It was calculated based on differences in DASS-21 categories reported in a previous study [38], using the most conservative estimates for mean difference and standard deviation. The calculation was performed using G*Power software version 3.1.9.2, with parameters set at a 5% significance level (α-error), 95% study power (β-error), and a two-tailed test. The minimum required sample size was determined to be 480 participants. To account for possible non-responses or incomplete data, a 10% margin was added, setting the target sample size at 528. However, a total of 715 responses were ultimately collected due to a higher-than-anticipated response rate.

### Instrument

The questionnaire was provided in English and Arabic to ensure clarity and avoid any language-related misunderstandings. The Arabic version, which was validated and used in previous studies [11, 14, 39], was included because Arabic is the participants’ mother tongue. The identity of the participants was undisclosed, and their participation was voluntarily with ability to withdraw from the study at any time. The online questionnaire was composed of two parts: demographic characteristics and the DASS-21 (Depression, Anxiety, and Stress Scale).

The first section of the questionnaire collected demographic and lifestyle information, which was stratified by the participants’ field of study (medicine or dentistry) for comparative analysis. The data included gender (male or female), marital status (single, married, or divorced), and parental status (no children, 1–2 children, or 3 or more children). Living conditions were also documented, with options for living alone, with family, with relatives, or with friends. Academic level was categorized into preclinical and clinical stages for medical and dental students. Additionally, participants indicated their college region, categorized as Eastern, Middle, Northern, Southern, or Western Saudi Arabia. Sleep duration was reported in four categories: less than 4 h, 4–6 h, 7–8 h, or more than 8 h per night. Social media usage was measured in terms of daily hours, ranging from less than 2 h to more than 8 h. Smoking status was recorded as non-smoker, previous smoker, or current smoker, and participants were asked whether they engaged in regular physical exercise (yes or no).

The second part of the questionnaire utilized the DASS-21, a tool designed to assess three psychological dimensions: depression, anxiety, and stress. The validity of the DASS-21 has been well-established in various populations, demonstrating high internal consistency and convergent validity across numerous studies [22, 40, 41]. The DASS-21 comprises 21 items that are evenly distributed across the three subscales, each evaluating specific aspects of mental health. The depression subscale assesses feelings of worthlessness, lack of interest or pleasure (anhedonia), and low motivation. The anxiety subscale measures symptoms such as nervousness, autonomic arousal, and muscle tension. The stress subscale evaluates emotional strain, overreaction, and difficulty in staying calm or relaxed.

Each item in the DASS-21 is rated on a 4-point Likert scale: 0 for “Did not apply to me at all,” 1 for “Applied to me to some degree, or some of the time,” 2 for “Applied to me to a considerable degree, or a good part of the time,” and 3 for “Applied to me very much, or most of the time.” The scores for each subscale are summed and doubled to align with the original DASS-42 scoring system [42]. Based on the total scores, participants are classified into one of five severity categories for each subscale. For the depression subscale, the categories are: normal (0–9), mild (10–13), moderate (14–20), severe (21–27), and extremely Severe (28+). For the anxiety subscale, the categories are normal (0–7), mild (8–9), moderate (10–14), severe (15–19), and extremely severe (20+). For the stress subscale, the classifications are normal (0–14), mild (15–18), moderate (19–25), severe (26–33), and extremely severe (34+).

### Statistical analysis

Statistical analysis was utilized using IBM SPSS Statistics for Macintosh, Version 28 (SPSS Corp., released in 2021). Kolmogorov–Smirnov test was used for normality test. Mann–Whitney and Kruskal–Wallis tests were used for median comparisons tests. Frequency and percentages were used in descriptive statistics. According to Lovibond and Lovibond (1995), the three DAS domains were categorized into three categories in which each category has its specific range (stress: 0–28<, anxiety: 0–20<, and depression: 0–34<). Ordinal regression was performed to assess the association between predictor variables and each mental health outcome (stress, anxiety, and depression). Multicollinearity among predictor variables was assessed and found to be within acceptable limits, indicating that the regression models were not affected by multicollinearity. Moreover, the test of Parallel Lines was conducted to verify the proportional odds assumption required for ordinal regression. The results of the test were insignificant for all three outcome models (stress, anxiety, and depression). Alpha level of significance of tests were set at < 0.05. Cronbach’s alpha reliability tests were used to assess the internal consistency of the questionnaire items. Although proportional sampling between medical and dental students was not achieved, subgroup analyses still yielded valuable insights despite the voluntary participation imbalance.

### Reliability analysis

Cronbach’s alpha was calculated to assess the internal consistency of the DASS-21 subscales (depression, anxiety, and stress). A Cronbach’s alpha value of > 0.70 was considered indicative of acceptable reliability [42]. Cronbach’s alpha reliability for stress was excellent ($\:\alpha\:$ = 0.891), anxiety was very good ($\:\alpha\:$ = 0.872) and depression items was excellent ($\:\alpha\:$ = 0.904).

### DASS-21 questionnaire

The participants were asked to rate each statement based on how much it applied to them over the past week (Table 1).

**Table 1:** DASS-21 questionnaire

1. I found it hard to wind down	0 1 2 3
2. I was aware of dryness of my mouth	0 1 2 3
3. I couldn’t seem to experience any positive feeling at all	0 1 2 3
4. I experienced breathing difficulty (e.g., excessively rapid breathing, breathlessness in the absence of physical exertion)	0 1 2 3
5. I found it difficult to work up the initiative to do things	0 1 2 3
6. I tended to over-react to situations	0 1 2 3
7. I experienced trembling (e.g., in the hands)	0 1 2 3
8. I felt that I was using a lot of nervous energy	0 1 2 3
9. I was worried about situations in which I might panic and make a fool of myself	0 1 2 3
10. I felt that I had nothing to look forward to	0 1 2 3
11. I found myself getting agitated	0 1 2 3
12. I found it difficult to relax	0 1 2 3
13. I felt down-hearted and blue	0 1 2 3
14. I was intolerant of anything that kept me from getting on with what I was doing	0 1 2 3
15. I felt I was close to panic	0 1 2 3
16. I was unable to become enthusiastic about anything	0 1 2 3
17. I felt I wasn’t worth much as a person	0 1 2 3
18. I felt that I was rather touchy	0 1 2 3
19. I was aware of the action of my heart in the absence of physical exertion (e.g., sense of heart rate increase, heart missing a beat)	0 1 2 3
20. I felt scared without any good reason	0 1 2 3
21. I felt that life was meaningless	0 1 2 3

Rating scale

0 = Did not apply to me at all

1 = Applied to me to some degree, or some of the time

2 = Applied to me to a considerable degree, or a good part of the time

3 = Applied to me very much, or most of the time

## Results

A total of 715 students completed the survey, comprising 550 dental and 165 medical students. While this reflects participant responsiveness, the sample includes a disproportionate number of dental students. The majority were under 26 years old (*n* = 676, 94.5%), single (*n* = 650, 90.9%), and without children (*n* = 650, 95.8%). Most identified as middle-income (*n* = 284, 39.4%) and reported living with family (*n* = 644, 90.1%). A greater proportion was in the clinical dental phase (*n* = 434, 60.7%), with the southern region most represented (*n* = 288, 40.3%). In terms of lifestyle, 43.1% (*n* = 308) slept 4–6 h nightly, with shorter sleep durations more common among medical students. Daily social media use of 2–4 h was most frequent (*n* = 184, 33.5%) among dental students. Approximately 14.5% (*n* = 104) reported being smokers, and 65.2% (*n* = 466) reported regular physical activity (Table 2). Internal consistency for the DASS-21 was high across all domains: stress (α = 0.891), depression (α = 0.904), and anxiety (α = 0.872).

**Table 2:** Characteristics of the study sample for all participants and by study field

		ALL (*N* = 715)	Dental (*N* = 550)	Medical (*N* = 165)
Gender	Male	344 (48.1)	227 (41.3)	117 (70.9)
	Female	371 (51.9)	323 (58.7)	48 (29.1)
Marital status	Single	650 (90.9)	497 (90.4)	153 (92.7)
	Married	63 (8.8)	51 (9.3)	12 (7.3)
	Divorced	2 (0.3)	0.4	0
Having kids	No kids	685 (95.8)	528 (96.0)	157 (95.2)
	1–2 kids	24 (3.4)	20 (3.64)	4 (2.42)
	≥ 3 kids	6 (0.839)	2 (0.364)	4 (2.42)
Living condition	Alone	52 (7.38)	43 (21.1)	9 (5.5)
	With Family	644 (90.1)	493 (89.6)	151 (91.5)
	With Relatives	10 (1.4)	7 (1.3)	3 (1.8)
	With Friends	9 (1.3)	7 (1.3)	2 (1.2)
College level	Preclinical dental	116 (16.2)	116 (21.0)	-
	Clinical dental	434 (60.7)	434 (13.8)	-
	Preclinical medical	74 (10.3)	-	74 (44.8)
	Clinical medical	91 (12.7)	-	91 (55.2)
College region	Eastern	28 (3.9)	24 (4.4)	4 (2.4)
	Middle	175 (24.5)	167 (30.4)	8 (4.8)
	Northern	65 (9.1)	50 (9.1)	15 (9.1)
	Southern	288 (40.3)	193 (35.1)	95 (57.6)
	Western	159 (22.2)	116 (21.1)	43 (26.1)
GPA	Fair	31 (4.3)	19 (3.46)	12 (7.27)
	Good	173 (24.3)	122 (22.2)	51 (30.9)
	Very good	278 (38.9)	221 (40.3)	56 (33.9)
	Excellent	233 (32.6)	187 (34.1)	40 (27.9)
Sleeping hours	< 4 h.	43 (6.0)	21 (3.8)	22 (13.3)
	≤ 4–6 h. <	308 (43.1)	213 (38.7)	95 (57.6)
	≤ 7–8 h. <	227 (38.7)	250 (45.5)	27 (16.4)
	8 h. ≤	87 (12.7)	68 (12.4)	21 (12.7)
Using social media	< 2 h.	85 (11.9)	42 (7.6)	43 (26.q)
	2–4 h.	279 (39.0)	184 (33.5)	15 (9.1)
	5–6 h.	193 (27.0)	170 (30.9)	23 (13.9)
	6–7 h.	0 (0.0)	85 (15.5)	4 (2.4)
	8 < hrs.	89 (9.5)	68 (12.4)	0 (0.0)
Smoking status	Non-smoker	565 (79.0)	438 (79.6)	127 (77.0)
	Prev. smoker	46 (6.4)	31 (5.6)	15 (9.1)
	Current smoker	104 (14.5)	81 (14.7)	23 (13.9)
Doing exercise	No	249 (34.8)	365 (66.4)	101 (61.2)
	Yes	466 (65.2)	185 (33.6)	64 (38.8)

**Fig. 1:**
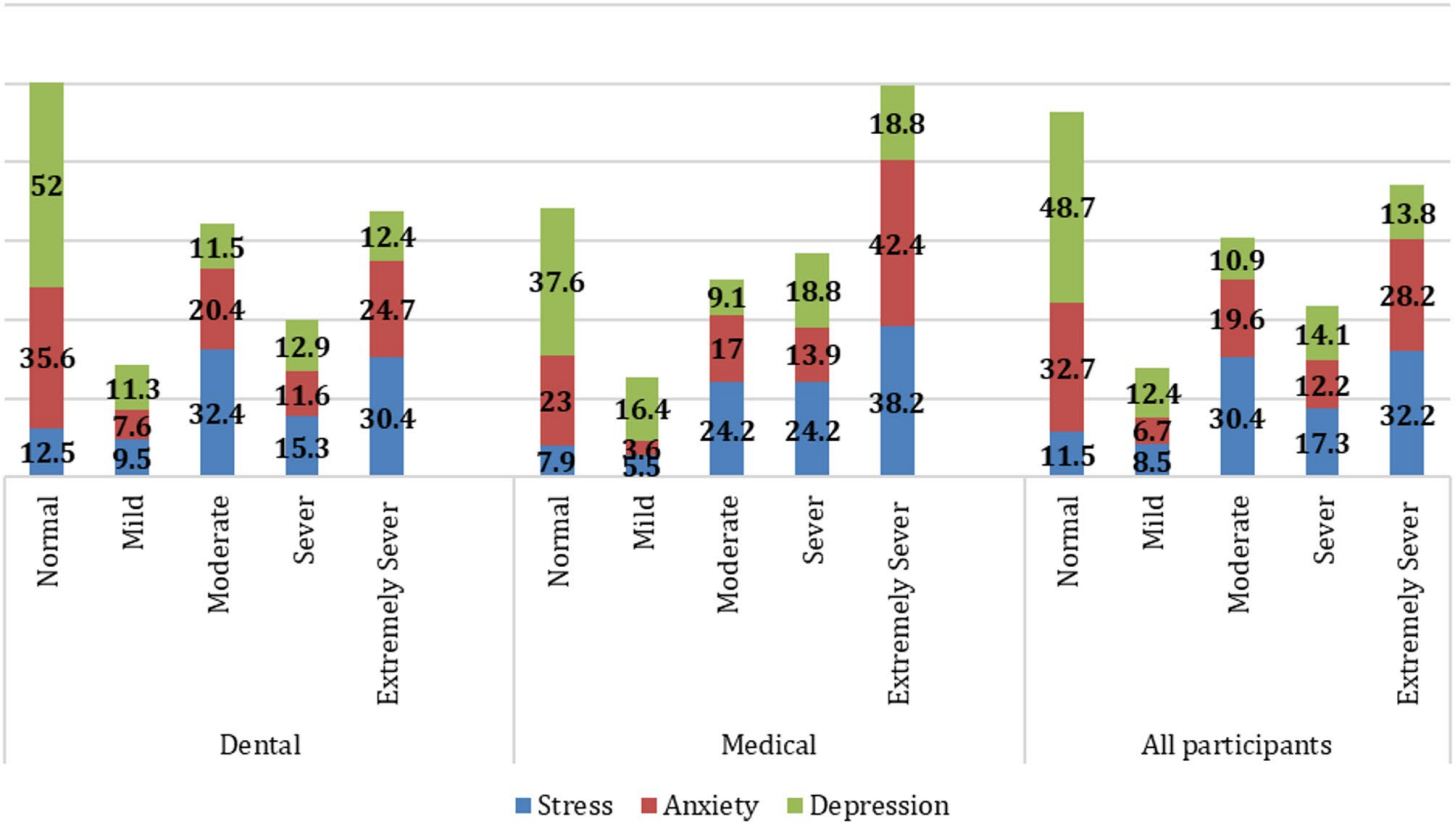
Percentage of DAS domain scores for all participants, stratified by field of study

Among all participants, stress was the most prevalent domain, with 32.2% classified as extremely severe. Medical students had a higher proportion in this category (38.2%) than dental students (30.4%). For anxiety, 28.8% were categorized as extremely severe, including 42.4% of medical students. Depression levels were more evenly distributed, with 18.8% of medical students classified as extremely severe and 48.7% of the overall sample reporting normal scores (Fig. 1).

Group comparisons showed no significant gender-based differences in stress (*p* = 0.410), anxiety (*p* = 0.964), or depression (*p* = 0.544). Living conditions influenced stress significantly (*p* = 0.027), with students living with friends reporting higher median scores. Medical students scored significantly higher than dental students across all DAS domains (*p* < 0.001 for stress, anxiety, and depression). Clinical-phase medical students reported higher stress, while preclinical dental students had higher anxiety and depression scores (*p* < 0.001 for all). Regional analysis showed significantly higher DAS scores in the northern and southern regions (Table 3). Academically, students with a “fair” GPA exhibited significantly higher stress and depression (*p* < 0.001). Anxiety was highest among students with an “excellent” GPA (*p* < 0.001). Students who reported sleeping fewer than 6 h had significantly higher DAS scores (stress: *p* < 0.001; anxiety: *p* = 0.006; depression: *p* = 0.001). Increased daily social media use was also associated with higher DAS scores across all domains (Table 3).

**Table 3:** Comparison of DAS domain scores among different sociodemographic variables for all participants (*n* = 715)

		Stress		Anxiety		Depression	
		Median (IQR)	*P*	Median (IQR)	*P*	Median (IQR)	*P*
Gender	Male	20.0 (14.0)	0.410	12.0 (18.0)	0.964	16.0 (20.0)	0.544
	Female	20.0 (16.0)		12.0 (16.0)		16.0 (18.0)	
Marital status	Single	16.0 (16.0)	0.295	6.0 (8.75)	0.744	8.0 (9.0)	0.621
	Married	22.0 (14.0)		6.0 (9.0)		8.0 (8.0)	
Having kids	No kids	20.0 (16.0)	0.314	12.0 (16.0)	0.285	16.0 18.0)	0.248
	1–2 kids	22.0 (17.5)		11.0 (17.5)		14.0 (19.5)	
	≥ 3 kids	22.0 (20.0)		2.0 (13.0)		8.0 (11.5)	
Living condition	Alone	18.0 (15.0)	**0.027**	12.0 (14.0)	0.524	14.0 (16.0)	0.545
	With Family	20.0 (16.0)		12.0 (18.0)		16.0 (20.0)	
	With relatives	25.0 (12.0)		19.0 (33.0)		14.0 (18.5)	
	With Friends	28.0 (17.0)		16.0 (50.0)		18.0 (23.0)	
Field	Dental	20.0 (14.0)	**< 0.001**	10.0 (14.0)	**< 0.001**	14.0 (18.0)	**< 0.001**
	Medical	24.0 (14.0)		18.0 (18.0)		18.0 (20.0)	
College phase	Preclinical dental	20.0 (15.0)	**< 0.001**	24.0 (14.0)	**< 0.001**	21.0 (6.0)	**< 0.001**
	Clinical dental	19.0 (14.0)		10.0 (14.0)		13.0 (36.0)	
	Preclinical medical	22.0 (10.00)		18.0 (17.5)		18.0 (18.0)	
	Clinical medical	26.0 (42.0)		14.0 (20.0)		14.0 (18.0)	
College region	Eastern	16.0 (10.0)	**< 0.001**	12.0 (19.0)	**0.007**	16.0 (14.0)	**< 0.001**
	Middle	18.0 (14.0)		8.0 (14.0)		12.0 (14.0)	
	Northern	20.0 (18.0)		12.0 (18.0)		14.0 (17.0)	
	Southern	22.0 (16.0)		14.0 (16.0)		18.0 (20.0)	
	Western	22.0 (12.0)		15.0 (16.0)		14.0 16.0)	
GPA	Fair	28.0 (20.0)	**< 0.001**	14.0 (22.0)	**< 0.001**	28.0 (24.0)	**< 0.001**
	Good	22.0 (18.0)		12.0 (15.0)		18.0 (20.0)	
	Very good	16.0 (14.0)		10.0 (14.0)		14.0 (16.0)	
	Excellent	24.0 (13.0)		16.0 (19.0)		16.0 (18.0)	
Sleeping hours	≤ 6 h.	22.0 (16.0)	**< 0.001**	14.0 (16.0)	**0.006**	18.0 (20.0)	**0.001**
	> 6 h.	18.0 (16.0)		10.0 (15.5)		14.0 (16.0)	
Surfing social media	≤ 4 h.	22.0 (16.0)	**< 0.001**	14.0 16.0)	**< 0.001**	18.0 (20.0)	**0.001**
	> 4 h.	18.0 (16.0)		10.0 (14.0)		14.0 (16.0)	
Smoking status	Non-smoker	24.0 (16.0)	**0.038**	14.0 (18.0)	0.138	18.0 (18.0)	0.623
	Prev. smoker	10.0 (10.5)		4.0 (10.5)		9.0 (12.5)	
	Current smoker	14.0 (10.0)		6.0 (8.0)		8.0 (12.0)	
Doing exercise	Yes	20.0 (14.0)	0.172	12.0 (14.0)	0.797	16.0 (16.0)	0.084
	No	22.0 26.0)		12.0 (22.0)		16.0 (24.0)	

The boldface entries indicate statistically significant values

Ordinal regression analysis (Table 4**)** revealed that students from the northern (OR = 8.30, *p* = 0.009), southern (OR = 2.76, *p* = 0.009), and middle (OR = 6.16, *p* = 0.019) regions had significantly higher odds of reporting stress compared to those from the western region. Lower odds of stress were observed among students using social media for ≤ 4 h daily (OR = 0.372, *p* = 0.003) and those with a very good GPA (OR = 0.041, *p* < 0.001). For anxiety, higher odds were associated with residence in the northern (OR = 6.89, *p* = 0.023) and eastern (OR = 7.14, *p* = 0.026) regions. No statistically significant associations were found for gender, field of study, or living status. Regarding depression, higher odds were observed among students from the northern (OR = 5.82, *p* = 0.044) and southern (OR = 4.92, *p* < 0.001) regions. Regular physical activity (OR = 0.657, *p* = 0.005) and sleeping fewer than 6 h (OR = 0.453, *p* = 0.016) were associated with lower odds of depression. All multivariable models met the proportional odds assumption. The results control for potential confounders across demographic, academic, and lifestyle factors.

**Table 4:** Ordinal regression analysis of factors associated with stress, anxiety, and depression levels

Variable	Category	Stress				Anxiety				Depression			
		OR	P	95% CI		OR	P	95% CI		OR	P	95% CI	
Gender	Males	0.856	0.299	0.638	1.15	0.830	0.217	0.618	1.12	1.08	0.626	0.795	1.47
	Females	Reference	.	.	.	Reference	.	.	.	Reference	.	.	.
Marital status	Single	1.10	0.725	0.654	1.84	1.08	0.784	0.643	1.79	1.08	0.791	0.633	1.82
	Married	Reference	.	.	.	Reference	.	.	.	Reference	.	.	.
Living condition	Alone	0.544	0.407	0.129	2.30	2.18	0.255	0.571	8.32	0.618	0.486	0.159	2.40
	With Family	0.443	0.229	0.118	1.67	1.57	0.466	0.467	5.27	0.818	0.745	0.244	2.74
	With relatives	1.35	0.743	0.862	7.99	5.43	0.053	0.978	30.1	1.01	0.995	0.183	5.53
	With Friends	Reference	.	.	.	Reference	.	.	.	Reference	.	.	.
College phase	Preclinical dental	1.03	0.940	0.524	2.01	0.815	0.546	0.419	1.58	1.14	0.698	0.582	2.25
	Clinical dental	0.676	0.245	0.349	1.31	0.586	0.111	0.304	1.13	0.806	0.530	0.411	1.58
	Preclinical medical	1.26	0.484	0.661	2.40	1.53	0.196	0.804	2.90	1.05	0.885	0.553	1.99
	Clinical medical	Reference	.	.	.	Reference	.	.	.	Reference	.	.	.
College region	Eastern	4.72	0.062	0.923	24.1	7.14	0.026	1.27	40.0	4.75	0.087	0.799	28.3
	Middle	6.16	0.019	1.35	28.1	4.81	0.056	0.958	24.19	3.43	0.147	0.648	18.16
	Northern	8.30	0.009	1.71	40.2	6.89	0.023	1.30	36.5	5.82	0.044	1.05	32.46
	Southern	2.76	0.009	1.29	5.87	1.66	0.188	0.780	3.52	4.92	< 0.001	2.26	10.72
	Western	Reference	.	.	.	Reference	.	.	.	Reference	.	.	.
GPA	Fair	1.03	0.950	0.450	2.34	0.824	0.636	0.370	1.84	1.15	0.724	0.521	2.56
	Good	0.666	0.285	0.316	1.40	0.783	0.517	0.375	1.64	0.565	0.135	0.268	1.19
	Very good	0.041	< 0.001	0.008	0.203	0.087	0.005	0.016	0.474	0.101	0.010	0.018	0.578
	Excellent	Reference	.	.	.	Reference	.	.	.	Reference	.	.	.
Sleeping hours	≤ 6 h.	0.807	0.484	0.442	1.47	0.832	0.550	0.455	1.52	0.453	0.016	0.239	0.861
	> 6 h.	Reference	.	.	.	Reference	.	.	.	Reference	.	.	.
Social media	≤ 4 h.	0.372	0.003	0.193	0.717	0.666	0.219	0.348	1.27	0.570	0.104	0.289	1.12
	> 4 h.	Reference	.	.	.	Reference	.	.	.	Reference	.	.	.
Doing Exercise	Yes	0.898	0.457	0.676	1.19	1.16	0.527	0.687	1.21	0.657	0.005	0.491	0.880
	No	Reference	.	.	.	Reference	.	.	.	Reference	.	.	.

Reference group: Extremely severe

## Discussion

This study evaluated the prevalence and determinants of depression, anxiety, and stress (DAS) among dental and medical students, offering insight into psychological challenges in health profession education. The findings highlight the urgent need for tailored interventions to promote students’ mental well-being and academic performance.

### Overall psychological burden

This study identified notably high levels of DAS, with stress being most prevalent (88.5%; ES: 32.2%), followed by anxiety (67.3%; ES: 28.2%) and depression (51.3%; ES: 13.8%). Statistically significant differences were observed between dental and medical students.

While some earlier studies reported comparable anxiety and depression rates, the stress levels in this sample are the highest documented nationally and exceed most figures internationally [30, 43–47]. Pre-pandemic studies—such as Aboalshamat et al. (2015, 2017)—showed stress levels of 70.9% (ES: 9.5%) and 64% (ES: 6.7%) in Makkah and Jeddah, respectively [4, 43]. Mirza et al. (2021) reported lower stress prevalence (37.6%) among Saudi medical students, while Fawzy & Hamed (2017) reported 59.9% among Egyptian students [44, 45]. In contrast, post-pandemic or overlapping-pandemic studies showed higher distress, including Tripathi et al. (2022), who reported 72.1% stress (ES: 36.5%) among Saudi medical students [46], and de Oliveira Viana et al. (2024), who found 60.9% stress among Brazilian dental students [32]. This apparent post-pandemic escalation may stem from disruptions to clinical education, prolonged isolation, and diminished access to academic and peer support—factors that likely undermined students’ ability to cope and contributed to sustained psychological fatigue [47]. These challenges may have led to emotional disengagement, a loss of control, and increased vulnerability to long-term distress.

### Academic discipline differences

Medical students reported significantly higher DAS scores than dental students (*p* < 0.001), with a greater proportion experiencing extremely severe stress (38.2% vs. 30.4%), anxiety (42.4% vs. 24.7%), and depression (18.8% vs. 12.4%). Although the unequal distribution of medical and dental participants warrants cautious interpretation, these findings align with those of Tripathi et al. (2022) and others, which report a greater psychological burden in medical cohorts [46, 48, 49]. The intensity of medical training—characterized by dense curricula, clinical responsibility, and limited recovery time—may contribute to this disparity [50]. Stigma around help-seeking, fear of being perceived as weak, and concerns about confidentiality can prevent medical students from accessing support [51]. According to Torales et al. (2025), systemic barriers like lack of confidentiality and stigma limit the effectiveness of mental health services [52]. To address this, universities should integrate structured mental health services into medical education, fostering a culture of openness and trust.

### Transition stress and clinical exposure

Stress levels were significantly higher among clinical medical students (median: 26.0) compared to preclinical peers (22.0), echoing patterns reported by Dahlin et al. (2005), Kulsoom & Afsar (2015), and Makki et al. (2021) [1, 53, 54]. Stress during the clinical phase can be attributed to the abrupt shift in roles and responsibilities, including fear of causing harm, insufficient preparation, and ambiguous expectations from supervisors. A study conducted in the Middle East emphasized that lack of orientation, performance anxiety, and supervisory pressures were key sources of psychological strain during the early stages of clerkship training [55]. These findings suggest that clinical training programs should integrate structured orientation, clear role definitions, and emotional preparedness workshops early in the clinical phase to ease transition, reduce ambiguity, and promote psychological resilience among students.

### Early-Phase distress in dental education

Preclinical dental students showed significantly higher levels of stress, anxiety, and depression (*p* < 0.001 for all), with notable median differences in anxiety (24.0 vs. 10.0) and depression (21.0 vs. 13.0). This is consistent with earlier Saudi findings, which identified second- and third-year students as particularly vulnerable due to preclinical skill demands, assessment pressure, and limited clinical exposure [4]. Other studies, however, found no significant differences across academic years [20, 35], suggesting that local institutional factors, such as curriculum pacing, support systems, or faculty-student engagement, may moderate psychological risk. To support vulnerable students, dental programs should enhance mental health awareness in early training phases, offer preparatory workshops, and facilitate early engagement with faculty mentors.

### Psychosocial and behavioural determinants

DAS levels did not differ significantly by gender, marital, or parental status, diverging from findings in some earlier reports [56, 57]. This supports the view that academic and institutional factors may exert a stronger influence on student well-being [34, 55, 58]. Notably, students living with friends reported higher stress, reaffirming the protective role of family and emotional support [59]. Sleep and social media behaviour were also significantly associated with DAS scores. Students with shorter sleep duration and higher screen time reported elevated distress (*p* < 0.001). However, multivariable analysis showed a paradoxical finding: students sleeping < 6 h had lower odds of depression. This could be due to underreporting, reverse causality, or confounding by coping behaviours. Poor sleep quality has been linked to impaired executive functioning and emotional regulation, contributing to anxiety and depressive symptoms [60–63]. Similarly, excessive social media use may heighten stress through digital fatigue, disrupted sleep, and exposure to unrealistic social comparisons [64]. Therefore, wellness initiatives should incorporate education on healthy sleep habits and responsible social media use to mitigate these risks.

### Academic performance and regional variation

Students with “fair” GPAs reported significantly higher stress and depression scores (28.0; *p* < 0.001), confirming earlier studies that link academic underperformance with psychological distress [4, 65, 66]. Self-doubt, academic pressure, and fear of future failure may intensify mental health challenges, especially in competitive environments. To mitigate risk, institutions should implement early-warning systems, pair struggling students with academic mentors, and normalize help-seeking as part of professional development. Psychological services should be integrated into academic support, not framed as crisis interventions alone. Regionally, stress was most pronounced in the southern and western areas of Saudi Arabia. While this may reflect a limitation in sample distribution, given the predominance of participants from the southern region, our findings align with those of Aboalshamat et al. (2017), who reported elevated DAS levels in western Saudi Arabia [43]. Socioeconomic inequality, educational resources, and geographic barriers may contribute to this variation.

### Implications for support and intervention

The findings of this study offer a comprehensive picture of the psychological burden affecting Saudi medical and dental students—particularly clinical-phase medical and preclinical dental subgroups. These data reinforce the necessity of targeted, evidence-based interventions across key academic transitions. Institutions should embed mental health promotion into educational policy through multi-tiered strategies: confidential counseling access, resilience training, time management skills, and peer support structures. Faculty and administrators must also play an active role in modeling healthy behaviours and fostering inclusive, stigma-free learning environments. By shifting from reactive to preventive mental health support, universities can equip future healthcare professionals not only with clinical competence, but with the emotional resilience needed for long-term success.

### Strengths of the study

This study demonstrates several strengths that enhance its rigor and relevance. The use of the validated DASS-21 instrument ensured reliable measurement of depression, anxiety, and stress. A large and diverse sample drawn from various universities and regions across Saudi Arabia enriched the dataset and allowed for broader contextual insights. The inclusion of both preclinical and clinical students enabled meaningful subgroup comparisons, offering valuable perspectives on the influence of academic progression on psychological well-being. Additionally, the timing of data collection, conducted post-COVID-19, offers important insights into the pandemic’s lingering psychological impact on health profession students.

### Limitations of the study

This study has several limitations. Its cross-sectional design limits causal inference, and reliance on self-reported data may introduce bias through social desirability or inaccurate symptom reporting. The online format may have led to self-selection bias, attracting more motivated or distressed participants. The overrepresentation of dental students, likely due to investigator affiliation and institutional responsiveness, may reduce comparability between disciplines. Similarly, the predominance of participants from the southern region limits national generalizability. Differences in academic environments across universities may also have introduced unmeasured confounders. Although the DASS-21 is a validated screening tool, it is not diagnostic and lacks contextual sensitivity to distinguish between situational stress and clinical disorders. Finally, the absence of qualitative methods constrained deeper exploration of students’ lived experiences and coping mechanisms.

### Future directions

Future research should adopt longitudinal designs to clarify the causal pathways linking academic stressors to psychological outcomes in medical and dental students. Incorporating qualitative methods, such as interviews or focus groups, could provide richer insights into students’ personal experiences, perceived stressors, and coping behaviours. Multicenter studies across different countries and educational systems are needed to distinguish universal from culturally specific mental health determinants. Additionally, intervention-based research should assess the effectiveness of structured mental health initiatives—such as counselling services, resilience training, peer mentorship, and stress management programs—within academic institutions. Finally, future studies should consider emerging influences such as screen time, hybrid learning models, and long-term psychological effects of the COVID-19 pandemic on academic and personal functioning.

## Conclusion

This study highlights the high prevalence of depression, anxiety, and stress (DAS) among medical and dental students in Saudi Arabia, with clinical medical students and preclinical dental students exhibiting the highest levels of psychological distress. Using the validated DASS-21 instrument, moderate to severe symptoms were identified, reinforcing the urgent need for targeted interventions and institutional support to mitigate academic stress and promote mental well-being. By addressing a national gap in post-COVID-19 data, the study underscores the importance of collaborative efforts among educators, administrators, and mental health professionals to prioritize student psychological health. Future research should focus on designing and evaluating evidence-based strategies that foster resilience and create supportive academic environments for healthcare trainees.

## Supplementary Information

Supplementary Material 1.

## Data Availability

All data generated or analysed during this study are included in this published article.
