# Supplementary material for: Psychological well-being of medical and dental students in Saudi Arabia post worldwide pandemic: a cross sectional study
Source: BMC Med Educ. 2025 Sep 1;25:1238. doi: 10.1186/s12909-025-07817-0 (PMC12400703; doi:10.1186/s12909-025-07817-0)
Supplement: Supplementary file 1 — Supplementary Material 1. [file 12909_2025_7817_MOESM1_ESM.pdf]

May 2, 2021

**Scientific Research- College of Dentistry, Jazan University**	**Reference No.** CODJU-2118F
**Research Title:** Level of Depression, Anxiety and Stress Among Medical and Dental Students in Saudi Arabia.	**Date of Decision:** May 01, 2021
**Principal Investigator**: Dr. Ahmad Jabali	**Department:** RDS

The following item (√) have been received and reviewed in connection with the above study to be conducted by the above investigator.

(√) Application to conduct research project
(√) Research Proposal/Protocol
(√) Patient Information Sheet & Consent Form
(√) Questionnaire
(√) Investigator's CV

The committee's decision is :
(   ) Initial approved (waiting for Jazan University Ethical Approval)
(√) Approved
(   ) Modification required(item specified below or in accompanying letter)
(   ) Rejected (reasons specified below or in accompanying letter)

Comments: Investigator is required to:
1. Follow instructions, guidelines and requirement of the Scientific Research Unit- College of Dentistry, Jazan University.
2. Report any protocol deviation/violations/serious adverse events to the Research Unit.
3. Provide progress and closure reports to the Research Unit.

Kingdom of Saudi Arabia Ministry of Education University Jazan College of Dentistry

Dr. Wael Awadh
Scientific Research Unit Head
